# The European Green Deal — More Than Climate Neutrality

**DOI:** 10.1007/s10272-021-0963-z

**Published:** 2021-04-06

**Authors:** Sarah Wolf, Jonas Teitge, Jahel Mielke, Franziska Schütze, Carlo Jaeger

**Affiliations:** 1 Global Climate Forum e.V., Neue Promenade 6, 10178 Berlin, Germany; 2 Climate policy, German Institut for Economic Research (DIW Berlin), Mohrenstraße 58, 10117 Berlin, Germany; 3 Global Institute of Sustainability and Innovation, Arizona State University, Orchid House at the Brickyard, 21 E 6th St, Tempe, AZ 85287 USA

## Abstract

The European Green Deal aims at climate neutrality for Europe by 2050, implying a significant acceleration of emission reductions. To gain the necessary support, it needs to reduce regional and social inequalities in Europe. We present objectives in terms of jobs, growth and price stability to complement the emission reduction targets and sketch a proof-of-concept investment profile for reaching these goals. Substantial additional annual public investments, of about 1.8% of pre-COVID-19 GDP, are proposed for the next decade. Their allocation includes retrofitting the European building stock, consciously fostering a renewal of the European innovation system as well as complementary measures in the fields of education and health. The scenario outlined in this article is meant as an input to the urgently needed discussion on how the European Green Deal can shift the EU economy to a new development path that realises a carbon-neutral Europe by 2050 while strengthening European cohesion.

The European Green Deal (EGD) has been proposed as a mission for Europe to become the world's first carbon-neutral continent by 2050, and to strengthen European cohesion through this mission (von der Leyen, 2019). Both goals present massive challenges; we argue that they can be turned into not only an environmental, but also a social and economic opportunity.

**Sarah Wolf**, Freie Universität Berlin; and Global Climate Forum, Berlin, Germany.

**Jonas Teitge**, Zuse Institut Berlin; and Global Climate Forum, Berlin, Germany.

**Carlo Jaeger**, Arizona State University, Phoenix AZ, USA; and Beijing Normal University, China.

The target to cut greenhouse gas (GHG) emissions by at least 55% by 2030 (compared to 1990), proposed by the European Commission, has gained geopolitical weight with Chinese plans to peak carbon emissions in 2030 and reach carbon neutrality no later than 2060 (UN, 2020). A whole range of nations and regions has declared similar goals.

Over the last two decades, processes of divergence and polarisation have been unfolding in the EU and the eurozone (Gräbner et al., 2020; Algan et al., 2017). With the coronavirus pandemic and the measures taken to control its spreading, these processes have intensified. Expected growth rates for Spain, Italy and Portugal in 2020 are -11%, -8.8% and -7.6% respectively; the expected losses for Germany and Denmark are less severe, at -5% and -3.5% respectively (European Commission, 2020e). At the same time, the fiscal impulse in response to the crisis amounts to 8.3% of GDP in Germany and 5.5% in Denmark, whereas in Spain, Italy and Portugal it amounts to 4.3%, 3.4% and 2.4% respectively (Anderson et al., 2020). The EU recovery plan promises to mitigate these processes – as should the EGD.

A European climate strategy aiming at carbon neutrality by 2050 can only be successful if it shifts the economy to a new development path that generates broad social and political support early on. This means it needs to come with tangible improvements of living conditions for European citizens at large, across all regions and social groups. Grounded in a line of research about how climate policy can trigger a transition to a new growth path (Jaeger et al., 2011, 2015; Jaeger, 2012; Schütze et al., 2017), this article presents a feasibility check of such a shift.

A historical transition like the EGD cannot and should not be planned in detail all the way to 2050, nor is it sufficient to declare an ambitious 2050 goal. First steps need to be specified. In this sense, we sketch a proof-of-concept EGD investment profile for 2021-2030 and explore its potential consequences in terms of emissions, unemployment, growth and inflation. Analysing what it takes to achieve the strictest GHG emission reduction target of 60% (as proposed by the European Parliament) technically, economically and socially, the main findings would remain in place also for the 55% target.

The quantitative scenario outlined is meant as a contribution to further discussions on designing and implementing the EGD in its first decade. By 2030, key developments will need to be carefully documented and evaluated in order to apply what is learned in the following decades.

## Relevant emissions dynamics and the path towards a carbon-neutral Europe

Among the multitude of dynamics relevant for the EGD, the EU's GHG emissions can be rather reliably specified; as illustrated in Figure 1, the pattern shows variation around a linear trend. In the baseline year of current climate policy, 1990, GHG emissions of today's EU27 countries stood at 4,857 Mt $CO_2$ equivalent ($CO_2e$).[1] This includes $CO_2$ emissions and other gases like methane, calculated by $CO_2e$. By 2018, they had fallen to 3,764 Mt (with 3,055 Mt $CO_2$ emissions), i.e. the decline was about 39 Mt per year on average (Eurostat, 2020b).[2] A 60% reduction by 2030 implies a target of 1,943 Mt. This requires an annual decline in the order of 162 Mt, a massive break with the trend of past decades.[3] After 2030, two decades remain for reducing the remaining 1,943 Mt to zero, implying an average annual decline of 97 Mt. It is reasonable to expect that if the challenging 2030 target is reached, the EU will then be able to move towards climate neutrality in 2050.

With the pattern thus illustrated, emission dynamics fluctuate around three different speeds of linear decline: a phase of sluggish reduction for the past three decades, a breakthrough decade starting at the time of writing, and two decades for bringing the effort to completion. Once available, the EU27 figures for 2020 may seem on track with emission reductions required for the coming decade, but the economic recovery expected for 2021 is likely to increase emissions again,[4] leaving little time for achieving the 2030 goal.

Figure 1

**Climate-neutral EU27 by 2050 via a 60% greenhouse gas emission reduction by 2030**

in Mt $CO_2e$

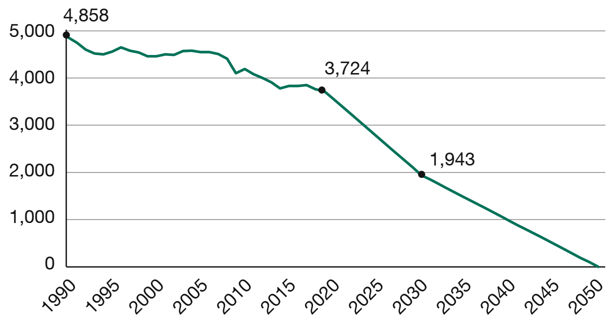

Sources: GHG Emissions 1990-2018 from Eurostat (2020b); 2019 own estimation following trend 1990-2018.

This is relevant for the role of carbon prices as they can quickly influence the way existing capital stocks and other durable goods are used. But they fail to incentivise the quick replacement of exisiting stocks necessary for carbon neutrality (Patt and Lilliestam, 2018). Therefore, direct regulation is as important as carbon prices, as shown by the rapid impact of recent EU emission rules for cars (Financial Times, 2020).

Neither carbon prices nor direct regulations, however, will automatically trigger the investments needed for building the infrastructure to support a carbon-neutral Europe. Public investment is therefore indispensable. At the same time, public investment is needed to overcome the widely underestimated slack in the EU economy (Brooks and Fortun, 2020). Because of this slack, well-targeted public investment can reduce GHG emissions without increasing costs for industries, but rather by creating additional output, employment and welfare – i.e. a new development path (Jaeger et al., 2011). The basic idea underlying this article hence is that the key to earning the buy-in of European citizens for the EGD lies in unleashing the full potential of the European economy by combining carbon

1 We use emissions data excluding international aviation.

2 At the time of writing, the most recent data available are those for 2018. The 2019 value is estimated by subtracting the average annual decline of 39 Mt from the 2018 figure, and the required reductions for 2020-2030 are computed from this.

3 The 55% target implies an annual decline of 140 Mt – hardly reducing the break with the past.

4 In the winter 2021 forecast, the EU Commission expected a GDP decline of 6.3% for the year 2020 as a whole and a rebound of 3.7% for 2021 (European Commission, 2020e).

taxes and direct regulation with EGD-oriented public investment.

## Further criteria for a new development path

Change required for carbon neutrality can be perceived as change for the better if the European Green Deal generates tangible and widely shared economic and social benefits. Along with the breakthrough in emission reductions discussed above, we consider the following additional criteria important:

*Unemployment rates below 7% and rates of youth unemployment below 15% across EU member states by 2030.* Overall unemployment in the EU (6.3% in the last quarter of 2019)[5] is not a sufficient criterion since unemployment figures for individual countries differ widely. Pre-COVID-19 levels ranged from 2% in the Czech Republic, 3.1% in Germany and 3.3% in Poland to 10% in Italy, 14.1% in Spain and 17.3% in Greece (Eurostat, 2020f).

Similarly, youth unemployment rates were 29.2% in Italy, 32.5% in Spain and 35.2% in Greece before the current crisis (Eurostat, 2020g) and are expected to rise significantly (Bacher and Tamesberger, 2020). The EGD can be experienced as an attractive perspective if it improves the current situation quickly, reaching and stabilising the stated goals by 2030.

*Strengthening convergence and cohesion*. A related issue is reducing inequality of wealth and income, both between and within European countries. The latter, in particular, has been increasing recently (Blanchet et al., 2019). Agreeing with European Parliament President David Sassoli that the "Green Deal must be an opportunity to fight inequality" (European Parliament, 2020), we leave the specification of measurable goals on this issue to further, urgent and promising research.[6]

*Average annual growth rates of EU GDP clearly above 2% until 2030*. The EGD should support a fast and strong recovery from the current crisis and help avoid another period of sluggish growth at a lower level than before the pandemic, as happened after the financial crisis. Between crises, growth rates hovered around 2% (European Commission, 2012; Trading Economics, 2019) and pre-COVID-19 growth estimates relevant for the decade 2020-2030 pointed to a range between 1.5% and 2% (Gros and Alcidi, 2013; European Commission, 2012; PwC, 2017; Knoema, 2019; Trading Economics, 2019). For the coming decade, a tangible improvement of the new development path over the previous one is needed. To the extent that improvements in other indicators of well-being – e.g. of public health, harmony with nature, fairness, fulfilling jobs – should materialise, such indicators may later become more salient than GDP growth (Stiglitz et al., 2018; Jakob et al., 2020).

*Inflation rates in line with price stability as defined by the ECB*, i.e. currently "below, but close to, 2% over the medium term" (European Central Bank, 2019).[7] Runaway inflation experiences are well documented at levels higher than 10%, but there is no evidence suggesting that a monetary policy focusing on a stabilisation goal of, say, 4% would be problematic, quite the opposite (Ball, 2014). However, an EGD driving inflation higher than the defined upper bound would be obstructed by the ECB raising interest rates. Most observers consider inflation rates since the financial crisis in the EU27 – averaging 1.55% between 2008 and 2019 (Eurostat, 2020c) – too low. Forecasts for the COVID-19 crisis are lower still: 0.7% for 2020 and 1.5% for 2021 (European Commission, 2020e). Inflation rates substantially below 2% are an indicator of slack and signal economic depression. The EGD should help to overcome this condition in the short run and avoid it in the long run.

## A proof-of-concept investment profile up to 2030

In view of the above criteria, for the first decade of the EGD, we consider the following annual expenditure profile, summarised in Table 1, building on two previous proposals (Wolf et al., 2020; Creel et al., 2020). The first items are geared directly towards emission reductions.

### A European electricity grid for 100% renewables

The electricity industry accounts for around 1,015 Mt $CO_2e$, or 27% of EU emissions (European Environment Agency, 2020b). Creel et al. (2020) describe the necessity and feasibility of frontloading investments into the European electricity grid for 100% renewables by 2050 (no fossil or nuclear fuels) described by the European Network of Transmission System Operators for Electricity (ENTSO-E, 2015). Their proposal amounts to an average of about €25 billion annually until 2030.

### Transforming the car-based mobility system

There were around 233 million passenger cars in the EU27 in 2018 (computed from the European Automobile Manu-

5 Current estimates see the COVID-19 pandemic leading to a rise in EU unemployment to around 7.7% in 2020 and around 8.6% in 2021 (European Commission, 2020a).

6 European Commission (2015) may be a good starting point.

7 For a discussion on inflation targets, as part of the ongoing strategy revision of the ECB, see Bremus et al. (2020).

Table 1
**Annual EGD investment streams for 2021-2030, emission reductions and main further effects**

| Share (rounded) | billion euro | Field | Mt $CO_2e$ | Societal benefits |
|---|---|---|---|---|
| | | Emission reduction trend preceding the EGD | 39 | |
| 10% | 25 | Frontloading a 100% renewables grid | 30 | |
| 8% | 20 | Transforming the car-based mobility system | 18 | Health |
| 8% | 20 | European Silk Road | 20 | Growth, cohesion |
| 27% | 70 | Energy renovation of buildings | 24 | Employment |
| 12% | 30 | R&D for energy-saving digitalisation | 30 | |
| 12% | 30 | Advanced green vocational education | catalyst | Convergence, employment |
| 12% | 30 | European breakthrough innovation system | catalyst | Employment |
| 4% | 10 | Subcontracting management tasks for EGD | catalyst | Employment |
| 8% | 20 | European planetary health policy | catalyst | Health, employment |
| 100% | 255 | Total public investment per year | | |
| | | Total emission reductions per year | 161 | |

Source: Authors' calculation.

facturers Association, 2019), responsible for about 15% of the EU27 $CO_2$ emissions (European Environment Agency, 2020b), i.e. 469 Mt $CO_2$, or about 2 t per car.[8] Currently, passenger transport uses almost 83% passenger cars, 9% motor coaches and under 8% rail (Eurostat, 2020e). Emissions can be reduced via more efficient cars and via shifting the modal split, e.g. increasing the share of public transportation, which is four times more efficient in terms of $CO_2$ than cars (UITP, 2014). The latter option can provide additional benefits in terms of noise, air quality, road safety and health. For cities, large variations in modal split – e.g. in London the share of cars is 10%, in Bilbao that of walking is 65% (EPOMM, 2020) – suggest possibilities for a sustainable mobility system.

For a breakthrough towards sustainable mobility (not only for cities), we consider an annual public investment of €20 billion reasonable, rounding up the infrastructure investments proposed by Wolf et al. (2020) for electric charging stations (€5 billion), cycling (€5 billion) and local public transport (€10 billion).

## A European Silk Road

Heavy duty trucks and buses were responsible for about 5.5%, or 208 Mt, of EU GHG emissions in 2018 (European Environment Agency, 2020b). As Creel et al. (2020) argue, it is time to balance the Chinese Belt and Road initiative with a European Silk Road initiative (see also Holzner et al., 2018; Mangalagiu et al., 2016). In terms of emissions, this offers a unique opportunity for a shift from yesterday's heavy vehicles to tomorrow's trains.

If the financing is organised through a special purpose vehicle (SPV) established by EU countries so as to be able to issue long-term bonds at near zero (positive or negative) real interest rates, public investment can be rather small compared to the overall volume. To set up the SPV and to ensure positive spillovers to the European transport network, an annual flow of €20 billion will suffice. The experience with the European Fund for Strategic Investments (EFSI), supporting investments of around €500 billion, using €33.5 billion of public investment and guarantees in five years, can serve as an example (European Investment Bank 2020).[9]

## Accelerating the energy renovation of the building stock

Buildings are accountable for 36% of the $CO_2$ emissions in the EU (European Commission, 2016a), presently about 1,100 Mt. The largest part of these emissions, about 750 Mt, is due to residential buildings.[10] With about 16 billion $m^2$ of residential floor space,[11] the average is about 47 kg/$m^2$.[12] Until now, only about 0.4% to 1.2% (European Commission, 2016b) of the building stock has been renovated each year, i.e. at most about 150 million $m^2$. At this pace, renovating the inefficient European building stock – esti-

8 European Commission (2020d) states 12%, but an average of about 160 g $CO_2$/km tailpipe emissions (ICCT, 2018) and an average of 12,000 km/year for the EU as a whole (Odyssee-Mure, 2020) is consistent with the former source.

9 For an analysis of EFSI-funded projects, see Schütze et al. (2020); unlike for EFSI, here the allocation of funds needs to be shifted away from funding airports and highways, e.g. to railways.

10 The calculation is based on relative energy intensity of residential and non-residential buildings as stated by Buildings Performance Institute Europe (2011).

11 Calculated by subtracting UK's 4 billion $m^2$ from a total EU28 floor space of 25 billion $m^2$ and taking into account that about 25% of the total floor space is non-residential (European Parliament, 2016a).

12 This is a very crude estimate obtained by dividing total emissions by total square metres. However, as Coma et al. (2019) find, data on the European building stock are scarce and spread over a factor of ten for their analysis of six European countries.

mated at about 75% of the total (European Commission, 2016a) – would take more than 60 years.

To accelerate energy renovation of residential buildings, we consider an annual investment in the order of €70 billion. Present estimates of energy renovation costs range from €200 to €450 per square metre, depending on the depth of renovation (European Parliament, 2016b). Providing €100 in grants per square metre out of €70 billion, on the condition that house owners invest at least €200 per square metre, would allow for the energy renovation of about 700 million m$^2$ per year.[13]

### Research and development in digitalisation for energy saving

While the information and communication technology (ICT) industry produces GHG emissions through its own energy use, digitalisation can lead to much larger emission reductions through energy savings – in buildings, transport, industry, households, agriculture and more. With the exception of some specialised niches, the EU ICT sector lags behind its main global competitors. In the EU, research and development (R&D) investment by ICT businesses was about 0.2% of GDP, and the amount publicly funded was less than 0.05% of GDP in recent years (European Commission, 2019). We suggest an additional public investment stream of €30 billion, i.e. another 0.2% of GDP, to change this situation in the direction of the EGD. These should be disbursed in such a way that they increase incentives for ICT companies to invest in applications promising large-scale energy savings and carry out the respective R&D in cooperation with building companies as well as regulating authorities.

While the following investments cannot be as directly associated with emission reductions, they are nevertheless essential for such reductions to happen under the given circumstances, e.g. to bring down emissions from industry.

### Advanced green vocational education

The EGD will disrupt occupational biographies in a number of sectors and requires innovative forms of advanced vocational training nurturing new skills on a broad scale (Jaeger, 2014). An annual investment stream of €30 billion can provide education opportunities, including a year of training for about 10% of the workforce in the construction sector (Wolf et al., 2020).

13 Grants should be disbursed on a first come, first served basis, with suitable regulations giving a strong signal to home owners that sooner or later their buildings will have to satisfy stringent energy standards.

### A European breakthrough innovation system

Since World War II, Europe has excelled in continuously improving existing technologies and practices, but not in generating breakthrough innovations. Catching up on such innovations developed elsewhere has worked occasionally, as in the case of planes and high-speed trains. However, for carbon neutrality, breakthrough innovations will be crucial to bring down the costs of transforming existing urban structures and in fields like green hydrogen, agriculture and ICT. In the view of the EGD, industry needs a combination of challenges from regulation and an overhaul of the European innovation system (see, e.g. Mazzucato 2016, 2018).

The US innovation system, which clearly has breakthrough capacity, differs in institutional arrangements and in funding magnitude:[14] US public R&D expenditures at the federal level alone (i.e. without states like Massachusetts or California) are in the order of 0.8% of GDP (Hourihan and Parkes, 2019). R&D expenditures of the EU are less than one-tenth of that – in 2019, e.g. the EU umbrella programme for R&D, Horizon 2020, amounted to around €12 billion, i.e. 0.075% of (then EU28) GDP. We hence suggest an additional investment stream of at least €30 billion, or about 0.2% of (now EU27) GDP, as a step in the direction of an innovation system capable of breakthroughs centred on the EGD.

### Subcontracting management tasks

Realising the EGD will require greatly amplifying the managerial capabilities of the EU and its member states. As in many other domains, public-private partnerships are important here. Carefully organised, an annual investment of €10 billion in human capital can become a unique experience of mutual learning by public administration and private enterprise for management at the local, national and European levels.

### A European planetary health policy

Finally, it was the COVID-19 crisis that triggered fundamental change in financial resources becoming available to tackle common challenges in the EU. To consolidate this process, it is vital to embed the goal of climate neutrality in the broader ambition of tackling the risks and seizing the opportunities of the Anthropocene. As a response to the coronavirus pandemic, the EU should develop a European planetary health policy (Whitmee and Haines, 2015). Initial tasks include building the capability

14 A discussion of an equivalent of the US Defense Advanced Research Projects Agency, with the EGD acting as a its backbone rather than the military is beyond the scope of the present paper (but see Marin, 2020).

and infrastructure for real-time monitoring of European health dynamics, initiating rapid large-scale contact tracking and testing, the cross-border transfer of medical equipment, vaccines and pharmaceuticals, step-wise development of health-conscious socio-ecological systems and expanding the global professional information exchange beyond intergovernmental channels.

Estimates for appropriate annual investments vary between €20 and €70 billion (European Commission, 2020c). As the long-term establishment of a European planetary health policy will be a stepwise process, we consider an initial budget of €20 billion per year, to be complemented by investments of member states.

The proposed investment profile amounts to an annual public investment of €255 billion, which is about 1.8% of the EU27's 2019 GDP and is in the range of volumes previously discussed in view of the EGD (European Commission, 2020b).

## Feasibility check

To show that the investment profile outlined can shift the EU economy to a new development path compatible with the proposed criteria, we roughly estimate plausible effects of the investments, based on literature and basic calculations. The obvious first step concerns emission reductions.

*The emission reduction trend before the EGD*. As the investments proposed here are in addition to existing measures, the average annual reductions of the past three decades (about 40 Mt of $CO_2e$ per year) form the basis on which to build. GHG emissions per capita differ widely among European countries, as does, e.g. carbon intensity in electricity generation – from Sweden's 13g $CO_2$/kWh to 773g $CO_2$/kWh in Poland (European Enviroment Agency, 2018). This leaves room for a continuation of the previous trend if countries can catch up to the least carbon-intensive ones.

*Frontloading of a 100% renewables European electric grid*. Presently, renewables in the EU make up 15% of total energy use, and the state of the grid is a key hindrance to rapidly expanding that share. Frontloading the 100% scenario proposed by the European Network of Transmission Operators for Electricity can deliver 30 Mt of annual emission reductions. As this corresponds to less than 3% of current emissions from electricity production, it is a conservative estimate.

*Transforming the car-based mobility system*. With tailpipe emissions of an average car of about 160g $CO_2$/km (ICCT, 2018) and around 122g $CO_2$/km for a new car in 2019 (European Environment Agency, 2020a), replacing an old car provides an efficiency gain of about 24%. Investments in charging infrastructure will be critical to achieving the 2020/2021 average fleet emission target of 95g $CO_2$/km (European Commission, 2020d), corresponding to about 40% gained. About 9 million old cars were replaced in 2018;[15] similar numbers lead to emission reductions of about 7 Mt if the greater efficiency gain is implemented. Further, we estimate that the investments outlined lead to a modal shift of 20% switching from passenger cars to an improved public transport system and 5% switching to biking and walking by 2030. Roughly, that corresponds to an annual modal shift of 2% and 0.5% respectively. Since each percent of the modal split using cars corresponds to 5.65 Mt of emissions,[16] this would save another 11.3 Mt, resulting in reductions of 18 Mt annually.

*The European Silk Road*. Today's railways are up to nine times less $CO_2$ intensive than the road (Finger et al., 2019), and a European Silk Road would constitute a "big push" for a European train-centred freight system of the future (Creel et al., 2020). According to CER (2015), a doubling of rail freight transport, with the freight shifted from roads, could result in a reduction of GHG emissions of around 45-55 Mt $CO_2e$ per year. As such an initiative takes time to realise its full potential, we estimate emission reductions in the order of 20 Mt $CO_2e$ per year.

*Accelerating the energy renovation of the building stock*. Turning an average inefficient building into an efficient building means reducing its emissions by around 75%,[17] or by an average of about 35kg $CO_2$ per square metre. Renovation of about 0.7 billion $m^2$ per year, as sketched above, then decreases emissions by about 24.5 Mt.

*R&D in digitalisation for energy saving*. Estimates for $CO_2$ emission reduction potential through ICT range from 0.1%-1.0% per year for households alone (Bastida et al., 2019) or to up to 3.7% across the whole society (British Telecommunications, 2019). The Global Enabling Sustainability Initiative (GeSI, 2015) estimates net GHG emission reductions in the order of 1.3% per year, corresponding to just under 50 Mt $CO_2e$ in the EU27's early 2020s. To exclude energy savings already accounted for above, we conservatively estimate 30 Mt $CO_2e$ per year.

15 In the EU27, 14.1 million cars were new (Eurostat, 2020d) in 2018, but the total number of cars increased by five million compared to 2017.

16 This is computed from the fact that 469 Mt correspond to 83% of the modal split.

17 Energy Class A compared to E, or D compared to A+ (Enev Oline, 2014).

Thus, the total annual emission reductions achievable with the proposed investment profile fit the requirements for the breakthrough decade 2020-2030, keeping in mind that investment streams into education, a breakthrough innovation system and a planetary health policy generally support these emission reductions.

Concerning the criterion of growth, €255 billion, slightly more than 1% of GDP, will lead to additional growth of at least 1.5% of GDP, due to a fiscal multiplier of at least 1.5 (Boitani and Perdichizzi, 2018). Together with the pre-crisis trend of around 1.5%, the annual growth rate will total at least 3%. According to the International Monetary Fund (2020), in conditions of high uncertainty, like those created by the COVID-19 crisis, the fiscal multiplier might even be larger than two, as the €255 billion would trigger further private investments. This does not consider important indirect effects, such as the role of technical change, nor the fact that training accelerates the rate of productivity growth (Sala and Silva, 2011), so that growth effects can be expected to be larger in the longer run.

The criterion of unemployment was defined at country level in view of the danger of divergence and the possibility of convergence in the EU. We consider the case of Greece, the EU country worst hit by unemployment, especially for youth. It has 2% of the EU population, about 800,000 unemployed in 2019 (Eurostat, 2020a) and a GDP of about €200 billion. Therefore, directing 5% of the €255 billion, i.e. about €13 billion, towards public investments in Greece would be a shock of about 6% of Greek GDP.

Under present conditions, employment would increase by about 1.2% over two years in response to a positive shock of 1% of GDP due to public investment (International Monetary Fund, 2020). Considering the resident working age population of about 3.8 million (Eurostat, 2020a), with the employment multiplier of 1.2, this shock translates to about 270,000 additional jobs over two years, constituting concrete steps towards the proposed targets, especially if hiring young people is incentivised.

Significant amounts of EGD public investments will need to be directed towards countries with excessive unemployment rates, but of course in such a way that countries near to or at full employment still experience a substantial stimulus. If the EU as a whole can accelerate growth to 3%, employment in Greece can further increase to meet the goals. By similar considerations, this also holds for other countries with unemployment rates currently above the stated goals.

At present, the main challenge for the ECB is to bring average inflation close to 2%. The investment push considered here would make this goal easier to reach, especially if wages catch up with productivity where they are lagging. The required increase of inflation would then facilitate an innovative dynamic of the overall economy, keep the danger of deflation at bay and create leeway for policy responses in future crises.

Conversely, to avoid a rise of inflation above the target set by the ECB, the quality of the European innovation system and the development of new forms of vocational education become crucial. Improvements in these areas create the capacity and flexibility needed to overcome bottlenecks that might lead to problematically high rates of inflation.

## A broader perspective

The present analysis is meant as an initial step in a broader research programme for understanding how Europe, and ultimately also other parts of the world, can shift to a new development path characterised by reducing emissions and increasing well-being.

Once such a new development path is reached, the level of public investment required to maintain it is likely to be lower than the level needed to trigger the transition. Given a decade of large-scale directed technical change, shifting back to a "brown" trajectory once a "green" one has been implemented is unlikely (Acemoğlu et al., 2012); the change in behaviour and social norms that leads to improved living conditions, such as cities with less pollution and noise (Gehl and Rogers, 2013) is also likely to remain.

It is setting in motion the virtuous circle of investment, well-being and innovation towards such a new development path that requires further research on potential options and development of the relevant technologies. Dialogue processes with decision-makers and citizens for deliberating what is desirable in a given context and negotiating contributions to the necessary changes are just as important (Mielke et al., 2017). Against this background, a co-evolutionary development of policies, technologies, cultural values and economic institutions seems to offer the best chance of successfully designing and implementing the European Green Deal.

The structure of a Green Deal outlined here is intended as a proof of concept. It aims to encourage discussion of elements of the EGD and related orders of magnitude that can lead to a shift to the new development path indicated. It also points to questions that will need answering, such as how to create a European innovation system capable of the breakthrough innovations needed to decarbonise not only Europe but the world economy as a whole.

A related and even more daunting challenge lies in establishing a common will to realise the European Green Deal across EU member states. The nationally fragmented initial response to COVID-19 has confirmed the need for reversing the trend of decreasing European cohesion, observable in rising support for anti-European parties in many countries, with Brexit as its most obvious example. It remains to be seen whether and how the ambitious mission of becoming the first climate-neutral continent may be turned into an opportunity for uniting the European people.
